# Retraction of: Navigating to support: experiences of forced migrant survivors of sexual and gender-based violence

**DOI:** 10.1093/eurpub/ckae076

**Published:** 2024-05-07

This is a retraction of: A Perez Aronsson, H Bradby, A Sarkadi, S Akyuz, G Warner, Navigating to support: experiences of forced migrant survivors of sexual and gender-based violence, *European Journal of Public Health*, Volume 32, Issue Supplement_3, October 2022, ckac130.178, https://doi.org/10.1093/eurpub/ckac130.178

On 25 October 2022, the conference abstract entitled ’Navigating to support: experiences of forced migrant survivors of sexual and gender-based violence’ was published within an issue supplement of the *European Journal of Public Health*.

The preliminary findings reported in the conference abstract were from secondary analysis. When preparing for manuscript publication the authors discovered that full documentation for the original data collection could not be produced. As a result, the authors have requested for the conference abstract to be retracted.

The authors have advised that they were not aware of the issue at the time of the conference and apologise for any inconvenience to the readers of the journal.

